# Discovery and Characterization of Diazenylaryl Sulfonic Acids as Inhibitors of Viral and Bacterial Neuraminidases

**DOI:** 10.3389/fmicb.2017.00205

**Published:** 2017-02-15

**Authors:** Anja Hoffmann, Martina Richter, Susanne von Grafenstein, Elisabeth Walther, Zhongli Xu, Lilia Schumann, Ulrike Grienke, Christina E. Mair, Christian Kramer, Judith M. Rollinger, Klaus R. Liedl, Michaela Schmidtke, Johannes Kirchmair

**Affiliations:** ^1^Department of Virology and Antiviral Therapy, Jena University HospitalJena, Germany; ^2^Centre for Chemistry and Biomedicine, Institute of General, Inorganic and Theoretical Chemistry, University of InnsbruckInnsbruck, Austria; ^3^Department of Pharmacognosy, Faculty of Life Sciences, University of ViennaVienna, Austria; ^4^Center for Bioinformatics, University of HamburgHamburg, Germany

**Keywords:** influenza virus, *Streptococcus pneumoniae*, coinfection, neuraminidase, small molecule inhibitor, diazenylaryl sulfonic acid

## Abstract

Viral neuraminidases are an established drug target to combat influenza. Severe complications observed in influenza patients are primarily caused by secondary infections with e.g., *Streptococcus pneumoniae*. These bacteria engage in a lethal synergism with influenza A viruses (IAVs) and also express neuraminidases. Therefore, inhibitors with dual activity on viral and bacterial neuraminidases are expected to be advantageous for the treatment of influenza infections. Here we report on the discovery and characterization of diazenylaryl sulfonic acids as dual inhibitors of viral and *Streptococcus pneumoniae* neuraminidase. The initial hit came from a virtual screening campaign for inhibitors of viral neuraminidases. For the most active compound, 7-[2-[4-[2-[4-[2-(2-hydroxy-3,6-disulfo-1-naphthalenyl)diazenyl]-2-methylphenyl]diazenyl]-2-methylphenyl]diazenyl]-1,3-naphthalenedisulfonic acid (NSC65847; **1**), the *K*_i_-values measured in a fluorescence-based assay were lower than 1.5 μM for both viral and pneumococcal neuraminidases. The compound also inhibited N1 virus variants containing neuraminidase inhibitor resistance-conferring substitutions. Via enzyme kinetics and nonlinear regression modeling, **1** was suggested to impair the viral neuraminidases and pneumococcal neuraminidase with a mixed-type inhibition mode. Given its antiviral and antipneumococcal activity, **1** was identified as a starting point for the development of novel, dual-acting anti-infectives.

## Introduction

Two of the major threats related to influenza A viruses (IAVs) are the genetic variability of the virus, which can lead to drug resistance (Hurt, 2014), and the lethal synergistic relationship (co-pathogenesis) that can form with bacteria, such as *Streptococcus (S.) pneumoniae* during superinfection (McCullers, 2014). At present, viral neuraminidase (NA) is the primary target of medicines for the treatment of influenza but only few inhibitors of this enzyme (NAIs) have been approved to date: oseltamivir, zanamivir, laninamivir, and peramivir (Li et al., 2015). Mutations of viral NA leading to drug resistance are often related to a loss of viral fitness, which limits the emergence of NAI-resistant strains (Abed et al., 2006; Ferraris and Lina, 2008; Nguyen et al., 2012). However, resistant strains can develop suddenly if compensatory mutations of NA enable efficient viral replication. This was observed, e.g., for oseltamivir in the season 2007/08 (Meijer et al., 2009; Baz et al., 2010; Bloom et al., 2010). In 2009, these oseltamivir-resistant seasonal H1N1 IAVs were succeeded by multiple reassorted pandemic H1N1 IAVs [A (H1N1)pdm09] (Hurt et al., 2009). These carry a NAI-sensitive NA gene of European swine influenza viruses (Bauer et al., 2012; Duerrwald et al., 2013), making them again susceptible to oseltamivir (Meijer et al., 2009).

The lethal synergism between (IAVs) and *S. pneumoniae*, which requires influenza virus prior to bacterial infection (McCullers and Rehg, 2002), has a grave medical and economic impact. Fatality rates for complicated bacterial pneumonia linked to this co-pathogenesis remain high, despite advances in medical care and the availability of a wide range of antibiotics as reviewed by McCullers and English (2008). These, authors state that “…the virus appears to worsen the clinical presentation, the tempo of progression, and the host immune response to the pneumonia.” They suggest that influenza viruses affect the pathogenesis and response to therapy. The contribution of a number of viral factors to the lethal synergism, in particular viral NA (McCullers and Bartmess, 2003; Peltola and McCullers, 2004; McCullers, 2014; Siegel et al., 2014), has been thoroughly investigated, whereas studies of bacterial factors have emerged only more recently. For example, we showed that influenza virus replication rates can be enhanced in the presence of bacterial NA in an *in vitro model*, and that inhibitors active against both viral and bacterial NA (Xu et al., 2008; Gut et al., 2011; Walther et al., 2015, 2016b; Grienke et al., 2016) can disrupt this synergism (Walther et al., 2016b). Therefore, the research of compounds with resistance-breaking capacity and dual activity on both viral and bacterial NAs represent promising and important avenues for the development of new anti-infective therapeutics.

Computational approaches have been playing a key role in the discovery and development of NAIs since the 1990's (von Itzstein et al., 1993). At that time, conformational flexibility of this target protein was generally not taken into account during the design of new inhibitors. Later the flexibility of NA received a lot of attention because of its impact on the geometric and electrostatic properties of the ligand binding site and hence on ligand binding. First indications of the remarkable flexibility of influenza NA came from crystal structures of group-1 NAs (N1, N4, N5, and N8) derived under different soaking conditions (Russell et al., 2006). It was found that the binding site-forming 150-loop of group-1 NAs is able to exist in at least two stable conformations. A more complete picture of the extent of this loop flexibility was developed by Amaro et al. (2007, 2009) who observed and characterized significant conformational shifts of the 150- and 430-loops by molecular dynamics (MD) simulations. It was suggested that this flexibility might also allow the binding of structurally distinct and in particular bulkier compounds. More recently, von Grafenstein et al. (2013) found that the dynamics of NA and specifically of the 150-loop are influenced by assembly states (i.e., viral NAs form homotetramers, while other NAs are active as monomers). Woods et al. (2013) investigated the unbinding and binding of oseltamivir using MD simulations, which confirmed the key role of the 150-loop.

Since the discovery of the conformational flexibility of the 150-loop, a larger number of NAIs have been reported that are thought or confirmed as binding to more open conformations of the enzyme. First examples of such inhibitors came from Cheng et al. (2008) who combined molecular dynamics (MD) simulation techniques with ligand docking in order to take protein flexibility into account during the virtual screening for NAIs. They screened the diversity set of the National Cancer Institute's (NCI) Developmental Therapeutics Program (DTP)^1^ on an ensemble of eight representative structures of N1, including an X-ray apo, an X-ray holo, and six frames extracted from MD simulation trajectories via clustering. In total, Cheng et al. (2008) selected 27 compounds, half of which would have been missed if only the X-ray structures would have been used for virtual screening. The activity of several of the compounds was detected with a fluorescence-based assay (FL assay) (Amaro et al., 2009). Further examples of small molecules that have been linked to binding to the open enzyme conformation include novel sialic acid analogs (Rudrawar et al., 2010; Adabala et al., 2013; Mohan et al., 2014) and natural products (Grienke et al., 2010, 2016).

Inspired by the work of Cheng et al. (2008) who identified inhibitors of viral NAs in the NCI DTP's Diversity Set, we extended this effort to screen the NCI DTP's complete molecular library with an ensemble-based virtual screening approach. Candidate molecules were acquired and tested *in vitro* on influenza and in addition on bacterial NA. Here we report on the key outcome of this work, the discovery of diazenylaryl sulfonic acids as dual inhibitors of influenza and *S. pneumoniae* NA.

## Materials and methods

### Virtual screening

The complete molecular library of NCI's DTP was downloaded from the institute's website^2^. The chemical structures were processed with Pipeline Pilot Student Edition (version 6.1.5, Accelrys, San Diego, CA): The “Organic Filter” and “Bad Valence Filter” components were used to remove unwanted molecules. A filter for the removal of potential pan assay interference compounds (PAINS) (Baell and Holloway, 2010) was not applied at that time to avoid loss of potentially interesting compounds (Senger et al., 2016). However, during later stages of the project, the RDKit PAINS 4 filter (Landrum, 2015) was applied to flag potential PAINS. All molecules were ionized at pH 6.5 using the “Ionize Molecule at pH” component (default settings). The 3D structures were minimized using the “Minimize Molecule” component (default settings). Docking of the ~134,000 compounds that passed all data processing steps was conducted with GOLD version 4.0 (Jones et al., 1997). Two X-ray structural models and 10 representative frames collected from MD simulations served as receptors. The X-ray structures were selected to represent the apo-structure (2hu0) and holo-structure (2hty) of N1 (open X-ray conformation). Full atom, explicit solvent MD simulations with Amber 10 (Case et al., 2008) were started from 2hty (chain B). The technical protocol of the MD simulations and extraction of representative frames from the MD trajectory is published in Grienke et al. (2010). In brief, 10 representative frames were selected for docking using a clustering algorithm from the 20 ns production phase of a full-atom, explicit solvent simulation.

The X-ray structural models were prepared for docking with the GOLD protein preparation wizard (default settings). Representative frames selected from the MD trajectories were used for docking (all water molecules were removed). GoldScore (part of the GOLD software package) was employed for scoring. Three docking runs were performed for each ligand and each protein conformation (with only the highest-ranked pose saved for hit selection).

### Compounds

The control compounds, oseltamivir carboxylate (GS4071; GlaxoSmithKline, Uxbridge, UK), the sialic acid analog 2,3-dehydro-2-deoxy-N-acetylneuraminic acid (DANA; Sigma-Aldrich, Deisenhofen, Germany) and imipenem (Sigma-Aldrich, Steinheim, Germany) were dissolved in bi-distillated water (10 mM). All test compounds were ordered from the NCI (Bethesda, MD) and dissolved in dimethyl sulfoxide (DMSO; Sigma Aldrich, Steinheim, Germany) to a final concentration of 10 mM. The stock solutions of all compounds were stored at 4°C until use. The purity of all compounds was checked by thin-layer chromatography (TLC) and high performance liquid chromatography (HPLC)—diode-array detector (DAD)—electrospray ionization interface (ESI)–mass spectrometry (MS) and determined as >95% in all cases.

Thin-layer chromatography (TLC) parameters: Mobile phase CH_2_Cl_2_-MeOH (3:1); stationary phase Merck silica gel 60 PF_254_; detected with staining reagents vanillin/H_2_SO_4_ at visible light, 254 and 366 nm UV light. HPLC-MS was performed on an Agilent 1100 s liquid chromatography (LC) system (Santa Clara, CA, USA) hyphenated to a Bruker-Daltonics Esquire 3000 plus ion trap. LC parameters: Stationary phase Phenomenex Hyperclone 5 μm C18 (150 × 3.0 mm); temperature: 40°C; mobile phase A = 80% water; B = 20% CH_3_CN; flow rate 1.0 mL/min; DAD detection wavelength: 210 nm; injection volume: 10 μL. Separations were performed by gradient elution (80/20 A/B in 15 min to 2/98 A/B) followed by a 5 min column wash (2A/98B) and a re-equilibration period of 10 min. All chemicals and solvents used were of analytical grade. MS parameters: Spray voltage 4.5 kV; sheath gas N_2_, 30 psi; dry gas N_2_, 6 L/min, 350°C; scanning range m/z 50–1500.

### Cell lines and viruses

Madin-Darby canine kidney (MDCK) cells (Cat.no. RIE 328, Friedrich-Loeffler Institute, Riems, Germany) were propagated as monolayer in Eagle's minimum essential medium (EMEM) supplemented with 10% fetal bovine serum (Life technologies, Darmstadt, Germany), 1% non-essential amino acids, 1 mM sodium pyruvate and 2 mM L-glutamine. Human lung carcinoma cells (A549; Institute of Molecular Virology, University of Munster, Germany) were grown in Dulbecco's Modified Eagle Medium (DMEM) supplemented with 10% fetal calf serum (PAA Laboratories GmbH, Cölbe, Germany). The HEK293 cell line (internal cell culture collection of the Department of Virology and Antiviral Therapy, Jena University Hospital, Jena) was cultured in serum-free EMEM supplemented with 2 mM L-glutamine.

H1N1 influenza viruses A/Jena/5258/2009 (Jena/5258; Kirchmair et al., 2011), A/Jena/8178/2009 (Jena/8178; Walther et al., 2016b), reverse genetics generated A/WSN/1933 (WSN/33) wild-type virus (Hoffmann et al., 2000) as well variants thereof containing specific amino acid substitutions in NA (Schade et al., 2015; Hoffmann et al., 2016), and the H_3_N_2_ influenza virus A/Hong Kong/68 (HK/68; Schaper and Brümmer, Salzgitter, Germany) were propagated on MDCK cells in serum-free EMEM supplemented with 2 mM L-glutamine, 2 μg/mL trypsin, and 0.1% sodium bicarbonate (test medium). Virus containing supernatant was harvested after about 48 h of incubation at 37°C, when the cytopathic effect (CPE) became microscopically visible. Aliquots were stored at −80°C until use. If not stated otherwise, the media supplements were obtained from Lonza Group Ltd. (Basel, Switzerland).

### Propagation of bacteria and preparation of precipitated pneumococcal total proteins

*S. pneumoniae* strain DSM20566 (serotype 1) was obtained from the German Collection of Microorganisms and Cell Cultures GmbH (Braunschweig, Germany). Bacteria were grown on Columbia blood agar plates (BD Biosciences, Heidelberg, Germany) at 37°C in 5% CO_2_ overnight or cultured in brain heart infusion (BHI) broth (Carl Roth GmbH + Co. KG, Karlsruhe, Germany) at 37°C as described elsewhere (Walther et al., 2015).

Precipitation of pneumococcal total proteins was described previously (Richter et al., 2015). Briefly, 200 μL of a pneumococci culture (grown overnight in BHI broth) were precipitated by 1.8 mL of ice-cold absolute ethanol (−20°C for 16 h). Aliquots were centrifuged at 3000 × g for 20 min at 4°C. The precipitate was washed with 1 mL of ice-cold 70% ethanol. After centrifugation at 3000 × g for 5 min at 4°C, the supernatant was removed and the pellet dried. The ethanol-precipitated proteins were dissolved in 50 μL of NA assay buffer and stored at −20°C until use.

### Expression and purification of *S. pneumoniae* neuraminidase constructs from *Escherichia coli*

Primers NA_116aaNdeI_fw (5′-TGCACGACATATGGAAAATGTC-3′) and NA_311aaNdeI_fw (5′-GTCAACATATGAAACGCTCAG-3′) were, respectively paired with NA_833aaXhoI_rv (5′-ATTGAAGGGCTCGAGCCTTG-3′) to generate two recombinant *S. pneumoniae* neuraminidase (NanA) variants with shortened C-terminus, namely NanA-LC (representing lectin and catalytic domains, residues 116–833) and NanA-CC (representing catalytic center, residues 311–833) constructs from individual *S. pneumoniae* strains DSM20566, CF8919, and CJ9400 (Xu et al., 2016). Briefly, each NdeI/XhoI double-digested polymerase chain reaction (PCR) product was ligated to the *Escherichia (E.) coli* expression vector pET-28a (Novagen, Merck KGaA, Darmstadt, Germany). The obtained plasmids encoded a series of N-terminal 6xHis-tagged NanA peptides. Transformation of *E. coli* was calcium chloride mediated. A 100 mL *E. coli* BL21(DE3) culture (Invitrogen life Technologies, Thermo Fisher Scientific, Darmstadt, Germany) with the plasmid was used to synthesize the enzyme. Gene expression was induced by isopropyl β-D-1-thiogalactopyranoside (IPTG) at a final concentration of 0.5 mM after a 4 h initial cultivation at 37°C. All enzymes were obtained from New England Biolabs (Frankfurt am Main, Germany). The broth was further incubated overnight at 25°C before harvested by centrifugation. The cell pellet was subjected to B-PER Protein Extraction Reagents (Thermoscientific, Darmstadt, Germany) for lysis and protein release. N-terminal His-tagged NanA was purified via HisPur Cobalt Spin Column (Thermoscientific, Darmstadt, Germany) and desalted by Pierce Concentrators PES 30K MWCO. The protein sample was stored in a 50% glycerol solution at −20°C.

### NA inhibition analyses

Viral (whole virus) and bacterial NA activity as well as their inhibition were measured by using a fluorescence (FL) assay with the substrate 2′-(4-methylumbelliferyl)-α-D-N-acetylneuraminic acid sodium salt hydrate (MUNANA, 100 μM; Sigma-Aldrich GmbH, Taufkirchen, Germany), and/or a hemagglutination (HA)-based NA inhibition assay (HA assay) with human erythrocytes (Institute of Transfusion Medicine, University Hospital Jena, Jena, Germany), both assays as published recently (Richter et al., 2015). Recombinant NanA (rNanA) and total precipitated bacterial protein of *S. pneumoniae* DSM20566 were used in FL and HA assays. Interference of the compounds with the FL signal (self-fluorescence and quenching) was excluded as described previously (Richter et al., 2015). The studied assay interference phenomena of test compounds in the HA assay included lysis or hemagglutination of the human erythrocytes by test compounds as well prevention of virus-induced hemagglutination (Richter et al., 2015). Each compound was tested at least three times in both NA inhibition assays.

### Enzyme kinetics

The Michaelis constant (*K*_m_), and the inhibition constant (*K*_i_) of NAIs were evaluated for WSN/33 variant NA-wt and rNanAs in FL assays (Marathe et al., 2013; Xu et al., 2016). No (control) or at least four inhibitor concentrations (as indicated in Supplementary Figures 1, 2) were applied in combination with four (NanA: 30, 60, 120, and 240 μM; viral NA: 15, 30, 60, and 120 μM) MUNANA substrate concentrations to determine the *K*_m_ and *K*_i_.

To study the inhibitory effect of NAIs on viral NA, whole virus (WSN/33 NA-wt) and NAI dilutions (each 10 μL) were preincubated for 20 min at 37°C or not pre-incubated before the addition of 30 μL of MUNANA substrate to see whether pre-incubation of NA with inhibitor affects activity (Barrett et al., 2011; McKimm-Breschkin et al., 2013). Relative fluorescence units (RFUs) correlating with the amount of 4- methylumbelliferone, the resulting cleavage product of MUNANA, were measured every 60 s for 60 min at 360/460 nm. In enzyme kinetics with rNanAs, RFU were measured after 5 min of incubation at 37°C. RFU were converted to 4-methylumbelliferone concentration in μM according to the 4-methylumbelliferone standard curve.

The Enzyme Kinetics Module of SigmaPlot 12.0 (Systat Software, San Jose, CA) was applied to fit the calculated velocity data (μM/min) to the Michaelis-Menten equation using nonlinear regression and to determine the *K*_i_ and *K*_m_ values of NAIs.

### Cytotoxicity and cytopathic effect inhibition assay

Cytotoxicity and CPE inhibition studies were performed on 2-day-old confluent monolayers of MDCK cells grown in 96-well plates as published (Schmidtke et al., 2001). Cytotoxicity was analyzed 72 h after compound addition. In the CPE inhibition assay, 50 μL of a serial half-log dilution of compound in test medium (maximum concentration 100 μM) and a constant multiplicity of infection (MOI: 0.003, 0.01, and 0.006 or 0.007 50% tissue culture infective dose (TCID_50_)/cell, for 8178/09, WSN/33, and HK/68, respectively) of test virus in a volume of 50 μL of the test medium were added to cells. Then, plates were incubated at 37°C with 5% CO_2_ for 48 h. Crystal violet staining and determination of the 50% cytotoxic (CC_50_) and 50% inhibitory concentration (IC_50_) was performed as described earlier (Schmidtke et al., 2001). At least three independent assays were conducted.

### Antibacterial assays

The antibacterial activity of the compounds was investigated using a microdilution assay in 96-well V-shape plates as well as biofilm assay in flat-bottom plates with *S. pneumoniae* DSM20566 as described earlier (Walther et al., 2015). Briefly, after cultivation on Columbia blood agar plates with 5% sheep blood at 37°C in an atmosphere enriched with 5% CO_2_ overnight, bacteria were grown to mid-exponential growth phase in BHI broth. For determination of bacterial growth and biofilm inhibition, samples of precultured pneumococci were diluted in BHI to match the turbidity of 1.5 × 10^8^ colony forming units (CFU) mL^−1^ (equivalent to a McFarland standard of 0.5). The spectrophotometric measurement at 565 nm was performed with a Den-1B McFarland densitometer (Grant Instruments, Cambridge, England). For the microtiter broth microdilution assay, untreated pneumococci in BHI (untreated control) or pneumococci with serial compound dilutions (dilution factor 2; maximum tested concentration 50 μM) were incubated in 96-well V-shape plates (Greiner bio-one GmbH, Kremsmünster, Austria) at 37°C with 5% CO_2_ for 18 h. The planktonic growth of untreated and compound-treated pneumococci was compared by measuring optical density (OD) at 620 nm. The biofilm inhibition assay was performed in 96-well F-bottom plates (Greiner bio-one GmbH, Kremsmünster, Austria) with pneumococci diluted in tryptic soy broth (TSB) for 2 h at 37°C with 5% CO_2_. After replacing the supernatant by TSB without (untreated control) or with diluted compound (dilution factor 2; maximum tested concentration 50 μM) and further 24 h of incubation, crystal violet staining, dye elution, and OD measurement at 550 nm were performed to quantify biofilm growth. In both antibacterial assays, the mean OD of six untreated controls was set 100% and used to calculate the 50% inhibitory concentration. At least three independent assays were performed.

In order to test whether the antibacterial concentrations of azo compounds exert a bactericidal or bacteriostatic activity, 100 μL of **1** and **2** (each 50 μM) were mixed with 50 μL bacteria (10^6^ cfu/mL *S. pneumoniae* DSM20566) in a 96-well V-shape plate in two parallels and incubated for 18 h at 37°C with 5% CO_2_. Thereafter, 100 μL of suspension from each well was sub-cultured on blood agar plates without test agent at 37°C with 5% CO_2_ to predict whether ≥99.9% of the bacteria were killed (i.e., a ≥3-log_10_ reduction in colony-forming units [cfu]/mL) on subculture. If there were <150 cfu per plate, the compound was rated bactericidal. In case of abundant bacterial growth (>150 cfu per plate), the compound was rated bacteriostatic. The antibiotic imipenem (Fitoussi et al., 1998; Walther et al., 2016a) served as control compound and was tested as described in parallel.

### Inhibitory effect of diazenylaryl sulfonic acids on viral replication in the absence and presence of pneumococcal NA

The effect of **1** (NSC65847) on virus replication in the absence and presence of rNanA of *S. pneumoniae* DSM20566 was investigated in A549 cells at 10 μM concentration (Walther et al., 2016b). The test medium contained DMEM, 0.5 μg/mL trypsin and 1.3% bicarbonate. The supernatant was harvested for determination of the inhibitory effect of test compounds on virus yield by plaque assay 48 h p.i. Two assays with two parallels were performed.

### Modified plaque reduction assay

The assay time and temperature conditions of the modified plaque reduction assay were described recently (Makarov et al., 2015). Here, confluent MDCK cell monolayers grown in 12-well plates were inoculated with ~30 pfu WSN/33 in 500 μL test medium. Cells and/or virus were exposed to **1** (100 μM) or oseltamivir (1 μM; for control) before infection, during the viral adsorption phase, after adsorption phase or during the whole replication cycle. Non-treated virus controls were included in all assays. Three independent assays were performed.

### Statistical evaluation

Mean and standard deviation (SD) values were analyzed using Microsoft Excel 2010. Statistically significant differences in *K*_i_ values and the NA inhibitory effect of A/WSN/1933 variants were analyzed by Welch's *t*-test (Microsoft Excel 2010).

## Results

### Identification of potential inhibitors of viral NAs by virtual screening

An ensemble of 12 structural models of influenza N1 was compiled that includes one X-ray apo-structure, one X-ray holo-structure and 10 representative snapshots from the MD simulation with the apo enzyme structure. The simulations (previously reported; Grienke et al., 2010) were run as full atom, explicit solvent MD simulations, and the representative conformations were extracted by clustering the conformations observed for a 20 ns trajectory of the production phase.

Subsequently, the NCI DTP's compound library of ~140,000 compounds was screened *in silico* with the docking software GOLD. For the purpose of virtual screening and hit selection, each of the 12 models was treated as individual target. We visually inspected the top-250 molecules of the individual hit lists and selected a total of 108 compounds that we deemed potentially active. GoldScore ranked all of the selected molecules among the top-30 positions of the individual hit lists. We ordered a subset of 80 compounds (prioritized by visual inspection) for biological evaluation. Of these, 73 compounds passed the purity and identity checks.

### Biological evaluation of compounds with fluorescence-based *in vitro* assays

**Figure 1:**
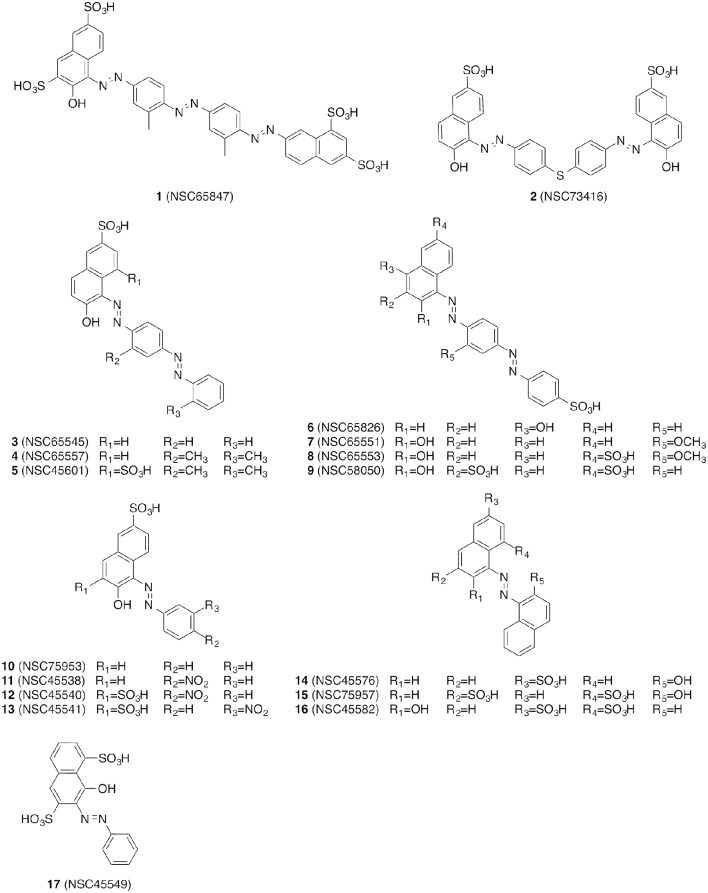
**Overview of the diazenylaryl sulfonic acids investigated in this work**.

The compounds were screened with a fluorescence-based *in vitro* assay on at least one influenza virus NA (i.e., A/Jena/5258/2009, A/Jena/8178/2009 or A/WSN/1933) and NA from *S. pneumoniae* strain DSM20566. Oseltamivir served as the control for the FL assay for which the measured IC_50_ values were between 0.3 and 3 nM for the various viral NAs and roughly 1 μM for NanA (Table 1). These values are in accordance with previously published data (Richter et al., 2015; Walther et al., 2015; Xu et al., 2016). Many of the compounds under investigation caused strong assay interferences, e.g., via self-fluorescence and fluorescence quenching, which affect FL assay results (results not shown) (Kongkamnerd et al., 2011; Chamni and De-Eknamkul, 2013; Richter et al., 2015). No significant inhibitory activity on influenza virus NA was observed for any of the tested compounds. However, for a diazenylaryl sulfonic acid (**3**), we were able to reliably detect and confirm biological activity against *S. pneumoniae* NA (IC_50_ = 11.62 μM; Table 1). We ordered analogs of **3** from the same source and characterized a total of 17 diazenylaryl sulfonic acids for which reliable assay results could be obtained (Figure 1 and Table 1). The most active compound, 7-[2-[4-[2-[4-[2-(2-hydroxy-3,6-disulfo-1-naphthalenyl)diazenyl]-2-methylphenyl]diazenyl]-2-methylphenyl]diazenyl]-1,3-naphthalenedisulfonic acid (NSC65847; **1**), had a measured IC_50_ < 1.5 μM for all of the three tested viral NAs and NanA. Three further compounds (**5**, **7**, **8**) inhibited the NA of WSN/33 with IC_50_ < 7 μM in addition to a distinct inhibition of the bacterial NA. In total, nine compounds (**1, 2, 4–8, 14**, and **16**) showed IC_50_ < 8 μM on *S. pneumoniae* NA. Among them is **14**, which Amaro et al. (2009) reported earlier as a weak inhibitor of influenza NA. We confirmed a weak activity for two of the three tested viruses.

**Table 1:** **Inhibition of IAV and *S. pneumoniae* NA in FL and HA assay**.

**Cpd**	**Code (NSC)**	**NA inhibition using FL assay^a^**				**NA inhibition using HA assay^a^**	
		**IC_50_ (μM)**				**MIC (μM)**	
		**IAV 8178/09**	**IAV WSN/33**	**IAV HK/68**	**S. p. DSM20566**	**IAV 5258/09**	**S. p. DSM20566**
1	65847	1.40 ± 0.39	0.44 ± 0.10	1.42 ± 0.44	0.32 ± 0.05	10.00 ± 0.00	7.26 ± 3.75
2	73416	Not active	20.73 ± 2.59	83.02 ± 5.67	1.85 ± 0.10	Not active^c^	10.00 ± 0.00
3	65545	Not active	Not active	Not active	11.62 ± 0.15	Not active^c^	Not active^c^
4	65557	84.27 ± 7.00	Not active	71.10 ± 22.01	4.79 ± 1.31	Not active^c^	Not active^c^
5	45601	16.00 ± 5.05	6.70 ± 0.43	17.79 ± 3.25	0.97 ± 0.30	10.00 ± 0.00	2.44 ± 1.25
6	65826	Not active	36.40 ± 4.20	83.45 ± 3.67	1.18 ± 0.55	24.40 ± 12.47	Not active^c^
7	65551	65.40 ± 24.22	4.25 ± 1.25	87.17 ± 8.03	5.60 ± 3.01	Not active^c^	Not active^c^
8	65553	Not active	6.51 ± 0.72	Not active	3.90 ± 0.51	Not active	Not active
9	58050	Not active	22.66 ± 2.91	Not active	31.96 ± 10.92	Not active	Not active
10	75953	Not active	Not active	Not active	10.18 ± 1.35	Not active^c^	Not active^c^
11	45538	Not active	33.07 ± 4.60	77.26 ± 10.57	23.19 ± 13.12	Not active	Not active
12	45540	Not active	Not active	Not active	Not active	Not active	Not active
13	45541	Not active	Not active	Not active	Not active	Not active	Not active
14	45576	Not active	28.92 ± 6.90	83.02 ± 6.25	5.74 ± 1.41	Not active^c^	Not active^c^
15	75957	Not active	28.73 ± 3.30	Not active	46.79 ± 5.27	Not active^c^	Not active^c^
16	45582	92.27 ± 10.01	44.27 ± 2.38	Not active	7.68 ± 0.98	31.60 ± 0.00	24.40 ± 12.47
17	45549	Not active	Not active	Not active	Not active	Not active	Not active
	Osel	0.003 ± 0.002	0.002 ± 0.001	0.0003 ± 0.0001	1.12 ± 0.32^b^	0.0007 ± 0.0004^b^	2.08 ± 1.25^b^

a *Mean and standard deviations of at least three independent experiments are shown*.

b *For comparison data published by Richter et al. (2015) were included here*.

c *Not active at concentrations that either did not provoke hemagglutination or prevented virus-induced hemagglutination of human erythrocytes at certain concentrations (Supplementary Table 1)*.

The low *K*_i_ values determined by a series of substrate-velocity curves against WSN/33 NA-wt (~0.6 μM) and pneumococcal NAs (0.11–0.19 μM) confirmed **1** as potential dual-acting NAI (Table 2). Significant differences in the measured *K*_i_ values were observed between the constructs including the lectin domain of NanA [9400_LC vs. 8919_LC (*p* = 0.016), and 9400_LC vs. 20566_LC (*p* = 0.024)]. In contrast, the construct consisting only of the catalytic domain (CC construct) was inhibited with equal effectiveness (*K*_i_ = 0.16 μM). The same trend was observed when testing DANA, a transition state analog inhibitor of influenza virus neuraminidase, albeit at higher concentrations (Xu et al., 2016).

**Table 2:** ***K*_m_ values for neuraminidases and MUNANA and *K*_i_ of 1**.

**Neuraminidases**		**Compound 1**		**Binding mechanism**
		*K*_m_^*^	*K*_i_ (μM)^*^	
*S. pneumoniae* recombinant NanAs	8919_LC	18.01 ± 2.39	0.11 ± 0.02	Mixed
	20566_LC	17.92 ± 2.12	0.12 ± 0.01	Mixed
	20566_CC	20.20 ± 4.74	0.16 ± 0.03	Mixed
	9400_LC	46.16 ± 1.71	0.19 ± 0.02	Mixed
	9400_CC	46.39 ± 3.93	0.16 ± 0.01	Mixed
Influenza virus A/WSN/33	without preincubation	40.00 ± 3.86	0.60 ± 0.06	Mixed
	20 min preincubation	44.17 ± 4.63	0.60 ± 0.17	Mixed

* *With the exception of Influenza virus A/WSN/33 without preincubation (n = 2), means and standard deviations of at least 3 assays are shown*.

### Confirmation of biological activity with a hemagglutination NA inhibition assay

An attempt was made to confirm the activity of these compounds under neutral pH and with cell surface sialic acids in the HA assay with human erythrocytes (Richter et al., 2015). Unfortunately, several of the tested compounds (i.e., **3**, **4**, **6**, **7**, **10**, **14**, and **15**) induced hemagglutination (Supplementary Table 1), rendering these compounds non-evaluable at certain concentrations in this cell-based NA inhibition assay. A further phenomenon observed was the inhibition of viral hemagglutinin-induced hemagglutination of human erythrocytes at 4°C by **2** and **5** using concentrations between 31.6–100 μM and 3.16–100 μM, respectively (Supplementary Table 1). Obviously, these compounds either bind to the surface of human erythrocytes or interfere with the viral hemagglutinin-sialic acid interaction. As in the FL assay, the strongest activities were measured for **1** (Table 1), which exhibited none of the aforementioned assay interferences. The trend for compounds to be more active on bacterial NA as observed with the FL assay was also confirmed with the HA assay.

### Inhibition profile of diazenylaryl sulfonic acids in influenza virus mutants

Six WSN/33 variants and wild-type (WT) virus generated via reverse genetics (Schade et al., 2014; Hoffmann et al., 2016) were used as a toolkit to examine the NA inhibition profile of diazenylaryl sulfonic acids in the FL assay. The highly active and virus-specific oseltamivir as well as the moderately active DANA with broad-spectrum activity were included as control compounds and studied for comparison. The inhibitory effect of the tested compounds was affected only very little by amino acid substitutions that confer resistance to oseltamivir (Table 3). However, H274Y led to a significant impairment in the activity of **1** as compared to the wild-type viral NA. A significant loss in activity was also observed for **5** when tested on WSN/33 NA variants carrying the H274Y, N294S, Q136L, or I427Q/M substitutions as compared to WSN/33 NA-WT. Surprisingly, Y155H led to a five-times decreased IC_50_ value for **2**, suggesting stronger binding to this mutant. The inhibitory effect of DANA was also affected only very little, but its inhibition profile differed from that of the diazenylaryl sulfonic acids.

**Table 3:** **Influence of amino acid substitutions in the influenza virus A/WSN/33 NA on the activity of test compounds**.

	**NA-wt**	**NA-H274Y**		**NA-N294S**		**NA-Y155H**		**NA-Q136L**		**NA-I427Q**		**NA-I427M**	
**Cpd**.	**IC_50_ μM^a^**	**IC_50_ μM^a^**	**FC^b^**	**IC_50_ μM^a^**	**FC^b^**	**IC_50_ μM^a^**	**FC^b^**	**IC_50_ μM^a^**	**FC^b^**	**IC_50_ μM^a^**	**FC^b^**	**IC_50_ μM^a^**	**FC^b^**
Oseltamivir	0.0018 ± 0.0005	0.45 ± 0.03^***^	248	0.15 ± 0.01^***^	83	0.012 ± 0.003^*^	7	0.0023 ± 0.0002	1	0.0061 ± 0.0015^**^	3	0.0029 ± 0.0003	2
DANA	2.44 ± 1.08	2.51 ± 1.38	1	8.12 ± 2.07^*^	3	8.36 ± 3.13^*^	3	2.33 ± 1.32	1	12.84 ± 0.75^***^	4	2.21 ± 0.62	1
**1**	0.44 ± 0.10	1.14 ± 0.13^**^	3	0.62 ± 0.13	1	0.33 ± 0.02	1	0.74 ± 0.17	2	0.51 ± 0.02	1	0.77 ± 0.29	2
**2**	20.73 ± 2.59	31.91 ± 1.13^**^	2	16.97 ± 1.47	1	4.79 ± 1.13^**^	0.2	35.38 ± 4.08	2	20.90 ± 4.21	1	26.31 ± 7.87	1
**5**	6.70 ± 0.43	18.04 ± 1.90^**^	3	20.13 ± 0.66^***^	3	6.06 ± 1.79	1	14.16 ± 0.26^***^	2	11.24 ± 1.57^*^	2	16.42 ± 2.12^*^	2

a Mean and standard deviation of 50% inhibitory concentration (IC_50_) determined in at least three independent fluorescence-based NA inhibition assay (Welch's t-test: ^*^p < 0.05,^**^p < 0.01,^***^p < 0.001).

b *Fold changes (FC) indicate the ratio of the mean IC_50_ of the influenza virus A/WSN/33 NA mutants and the IC_50_ of the wild-type (wt) NA*.

### Cytotoxicity, antiviral, and antipneumococcal activity

**Figure 2:**
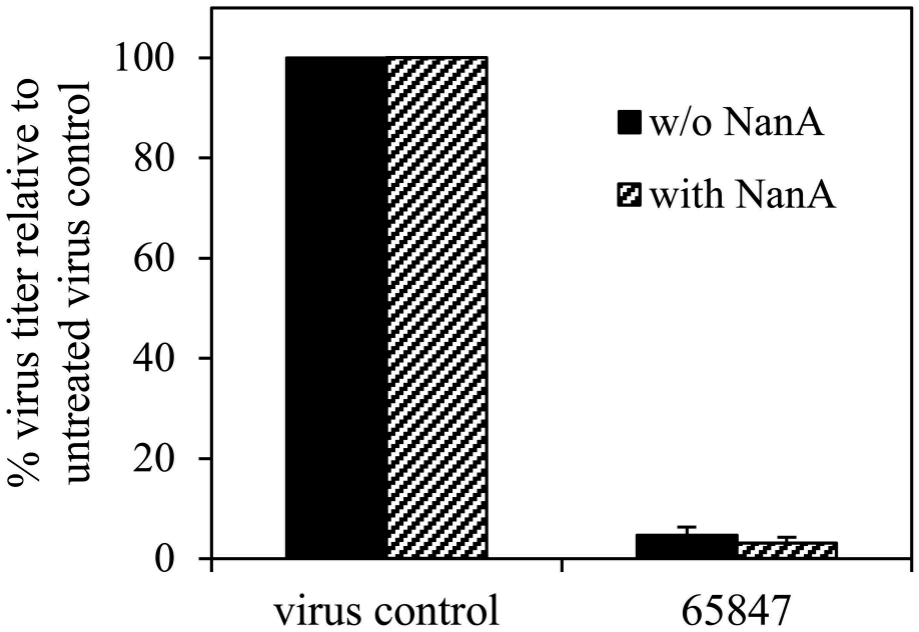
**Recombinant pneumococcal NA (NanA) does not reduce the inhibitory effect of 1 on virus yield**. Influenza virus A/Jena/8178/09 (Jena/8178)-infected A549 cells (MOI of 0.1 TCID_50_/cells) were treated with 10 μM of **1** in the absence or presence of NanA. Virus titers in pfu/mL were determined with plaque assay 48 h after infection. Virus control titer was set to 100% and inhibition of the control titer by NAI in % was calculated. Experiments were performed twice with two parallels. Results of one representative assay are shown exemplarily. Exact numbers of plaque-forming units are reported in Supplementary Table 2.

Cytotoxicity was ruled out for all compounds for concentrations up to 100 μM (Table 4). Six compounds (**1–4, 6**, and **7**) inhibited the 8178/09-, WSN/33-, or HK/68-induced cytopathic effect at IC_50_ < 15 μM. As we reported recently, NanA supports the spread and yield of 8178/09 in A549 cells (Walther et al., 2016b). Treatment with 10 μM of **1** significantly inhibited virus yield in both the absence and presence of NanA, underlining its dual activity (Figure 2 and Supplementary Table 2).

**Table 4:** **Cytotoxicity, antiviral, and antibacterial activity of studied compounds**.

**Cpd**.	**CC_50_ (μM)**	**50% inhibitory concentration and standard deviation (μM)^a^**				
		**influenza A virus**			***S. pneumoniae*** **DSM20566^b^**	
		**8178/09**	**WSN/33**	**HK/68**	**Planktonic growth**	**Biofilm formation**
1	>100	20.36 ± 2.51	7.17 ± 0.96	18.50 ± 9.59	28.75 ± 13.13	49%^b^
2	>100	9.09 ± 1.94	11.83 ± 3.19	6.19 ± 4.62	4.59 ± 2.45	47%^b^
3	>100	14.42 ± 8.16	52.20 ± 17.91	13.63 ± 3.18	20.14 ± 2.24	Not active
4	>100	12.10 ± 4.83	13.96 ± 2.68	13.19 ± 3.11	11.97 ± 6.81	46%^b^
5	>100	Not active	Not active	Not active	Not active	42.23 ± 2.57
6	>100	10.58 ± 0.71	24.22 ± 9.78	20.82 ± 1.99	6.14 ± 0.61	46%^b^
7	>100	3.28 ± 2.19	78.63 ± 16.18	4.22 ± 0.98	3.32 ± 0.72	46%^b^
8	>100	Not active	Not active	Not active	Not active	Not active
9	>100	Not active	Not active	Not active	Not active	Not active
10	>100	Not active	Not active	Not active	Not active	Not active
11	>100	Not active	Not active	Not active	Not active	Not active
12	>100	Not active	Not active	Not active	Not active	Not active
13	>100	Not active	Not active	Not active	Not active	Not active
14	>100	Not active	Not active	Not active	Not active	Not active
15	>100	Not active	Not active	Not active	Not active	Not active
16	>100	Not active	Not active	Not active	11.97 ± 6.81	46%^b^
17	>100	Not active	Not active	Not active	Not active	Not active
Oseltamivir		0.034 ± 0.022	0.009 ± 0.003	0.003 ± 0.001	Not studied^c^	Not studied^c^

a *At least three independent assays were used to calculate mean and standard deviation in the cytopathic effect inhibition assays in MDCK cells. The maximum tested concentration in the cytopathic effect inhibition and antibacterial assays was 100 and 50 μM, respectively*.

b *Inhibition of pneumococci activity at the maximum tested concentration of 50 μM*.

c *Not studied as control because confirmed as inactive (Walther et al., 2015)*.

The six antiviral compounds significantly reduced the planktonic growth of *S. pneumoniae* (Table 4). Confirmation of bacterial viability on blood agar plates revealed a bacteriostatic inhibition effect of the compounds (Supplementary Table 3). The biofilm formation was not or was only slightly affected (Table 4).

### Compound 1 abrogates virus infectivity

**Figure 3:**
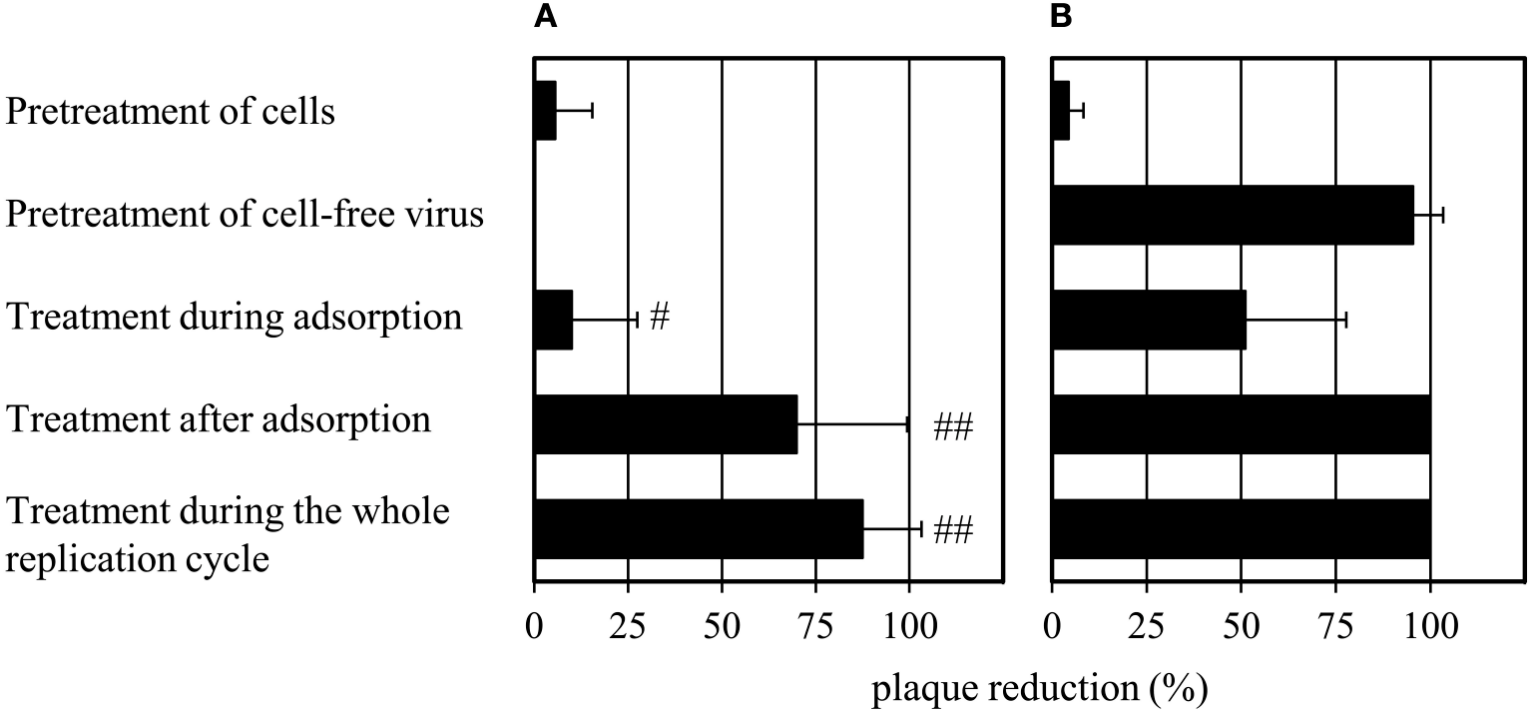
**Modified plaque reduction assay with (A)** 1 μM of oseltamivir and **(B)** 100 μM of **1**. “#” indicates a slight (plaques are half as large compared to the virus control) and “##” a strong (plaques have about 10% of the size of the virus control) plaque size reduction. Bars represent the mean values and SD of at least three experiments, each with two parallels per concentration. Images documenting the observed plaque size reduction are provided in Supplementary Figure 3.

The effects of **1** on the cell-free virus and different stages of the viral life cycle were evaluated and compared to that of oseltamivir (Figure 3). MDCK cells were treated with oseltamivir or **1** for 30 min at 37°C. The unbound compound fraction was removed by three washing steps before the addition of virus. Plaque numbers were not affected by pretreatment with oseltamivir or **1**. Surprisingly, pretreatment of the cell-free virus with **1** for 30 min at 37°C (compound dilution to no-effect concentrations before virus addition to MDCK cells) caused a ~95% reduction of plaque number. Oseltamivir was not active under these experimental conditions. It was also inactive when added during the 2 h of virus adsorption on MDCK cells at 4°C. In contrast, addition of **1** reduced the number of plaques by 50% during viral adsorption. When administering oseltamivir or **1** in the agar overlay after virus adsorption, the percentage of plaque reduction was enhanced. Administration of both compounds during the whole replication cycle (during and after adsorption) decreased plaque formation by up to 100%. Oseltamivir but not **1** strongly reduced the plaque size (Supplementary Figure 3).

## Discussion

The starting point of this work was the search for new inhibitors of viral NAs. During the course of the study we found that several diazenylaryl sulfonic acids picked up by virtual screening inhibited *S. pneumoniae* NA *in vitro* and that some analogs exhibited dual activity on viral and bacterial NAs.

Diazenylaryl sulfonic acids have been previously investigated in the context of influenza NA inhibition. For example, in Becht and Drzeniek (1968) reported on the inhibitory activity of the azo dyes congo red and trypan red against myxovirus NA (and viral replication), which they linked to a polyanionic effect. Shortly thereafter, Akerfeldt (Akerfeldt et al., 1971) reported on the antiviral (in particular also anti-influenza) activity of a larger collection of aromatic sulfonic acids. It was suggested that these compounds might be absorbed by the virions, thus blocking virus entry into the host cells. Diazenylaryl sulfonic acids also appear in the more recent literature, such as in the report of Cheng et al. (2008) of a virtual screening campaign on influenza NA using multiple structural models to represent the conformational flexibility of the target protein and in a related patent application (Amaro et al., 2009). With remazol brilliant blue R (an anthraquinone dye), a further compound with an aryl sulfonic acid scaffold was recently reported as an inhibitor of influenza virus NA (Hsu et al., 2013). To the best of our knowledge, there have been no reports of systematic efforts to characterize the activity of these compounds yet.

The compounds investigated in the current study contain substructures that have been associated with pan-assay interference (Baell and Holloway, 2010). Therefore, extra care was taken when conducting assays and interpreting their readouts. However, the presence of an azenylaryl acid structure in several established and well-tolerated aminosalicylate drugs (e.g., sulfasalazine and olsalazine), used for the treatment of chronic inflammatory diseases, demonstrates that the diazenylaryl scaffold could serve as a valid starting point for drug development. Intrigued by the reoccurring reports on (diazenylaryl) sulfonic acids inhibiting influenza virus NA in absence of conclusive evidence regarding their mode of action and the fact that our screening campaign also picked up this type of compound, we decided to investigate this phenomenon in detail.

The data compiled during this study indicate that diazenylaryl sulfonic acids can target influenza virus NA and *S. pneumoniae* NanA. Assay interference in NA inhibition assays was carefully ruled out by the concerted use of control measurements e.g., self-fluorescence, quenching of fluorescence and self-hemagglutination. Moreover, two different assays (FL and HA assays) as well as WSN33 NAs with single amino acids substituted in proximity of the active site and different recombinant pneumococcal NAs were used to confirm NA inhibition. Whereas, the FL assay is performed with the synthetic substrate MUNANA at acid pH, the HA assay allows testing of NA inhibition under more physiological conditions (cleavage of cell surface sialic acid at neutral pH) (Richter et al., 2015). The FL assay showed an inhibition potential of diazenylaryl sulfonic acids sensitive to the NA context: Activity of diazenylaryl sulfonic acids tended to be higher on bacterial NanA (corresponding to lower IC_50_ and *K*_i_ values) as compared to viral NAs. The bioactive chemical space of diazenylaryl sulfonic acids seems to be well defined and narrow for viral NAs but broad for pneumococcal NAs. The four compounds that inhibited at least one of the three tested viral NAs and pneumococcal NA all comprise one (**5**, **7**, **8**) or two (**1**) additional diazenylaryl units linked with their diazenylaryl sulfonic acid core. Compound **1** can be regarded as a dimer-like analog of the initial hit, **3**. None of the eight tested compounds that consist of a single diazenylaryl unit were active against viral NA but three of them were active against pneumococcal NA (**10**, **14**, and **16**). Thus, the dual activity toward both pneumococcal and viral NA seems to be associated with the diazenylaryl sulfonic acid core in combination with at least one additional diazenylaryl moiety. Unfortunately, only a minority of the tested diazenylaryl sulfonic acids was evaluable with the HA assay due to assay interferences. However, the obtained data fully confirm NA inhibition for oseltamivir and the two most active compounds, **1** and **5**. In addition, some significant differences in the inhibitory activity of compounds were observed against the mutated WSN/33 NAs (Table 3). The amino acid substitutions did not result in changes in IC_50_ values as large as the change observed for oseltamivir in the context of the H274Y mutation but were in a range comparable to that of DANA (although the pattern was different; Table 3).

**Figure 4:**
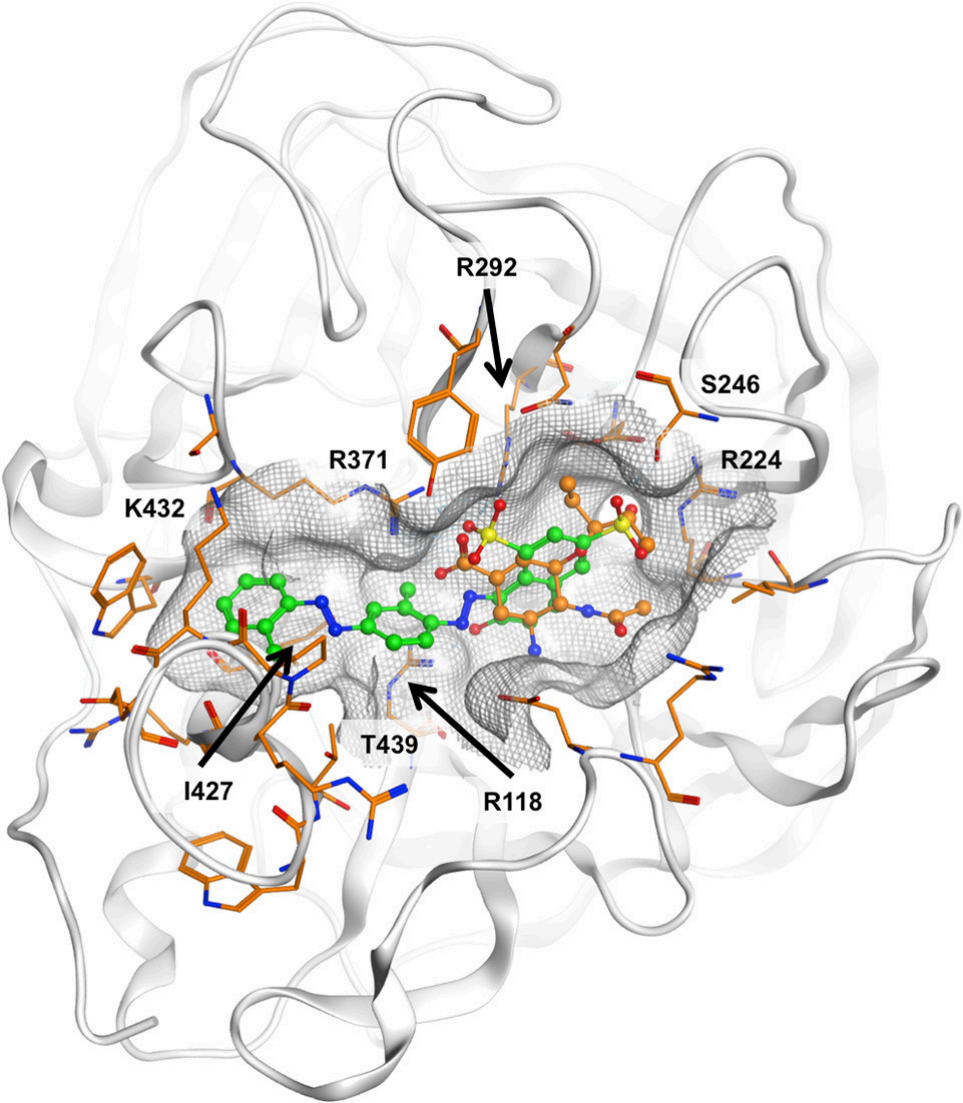
**Potential binding mode of 5 (green carbon atoms) to N1 neuraminidase [Protein Data Bank (PDB) 2hu0]**. Oseltamivir is depicted in ball-and-stick mode with orange carbon atoms. In the proposed binding mode, the sulfonic acid and phenolic moieties of 5 are oriented toward the positively charged R118, R224, R292, and R371, hence forming ionic interactions and a tight network of hydrogen bonds. The compound fits well to the molecular shape of the ligand interaction site. Hydrophobic surface contacts are formed, e.g., between the ligand's terminal phenyl moiety and I427 and K432.

The ligand binding sites of NAs have a highly polar character, with a large number of charged amino acids, such as arginines present. All approved NAIs comprise a carboxylic acid function that forms a salt bridge with R371. It is plausible that the sulfonic acid moiety that is present in all compounds under investigation could mimic this interaction. However, deriving the potential binding mode of diazenylaryl sulfonic acids proved difficult. Automated ligand docking using the X-ray structural models and the representative frames extracted from the MD trajectory did not consistently point to a specific ligand orientation. Manual docking of **5** into the substrate binding site of N1 NA resulted in a docking pose which we consider plausible because of good compatibility of chemical features and the molecular surfaces with the open and closed conformations observed in crystal structures (the open conformation is visualized in Figure 4). However, this binding mode does not explain the activity of **5** on pneumococcal NA. Apparently, this compound does not fit into the binding pocket of pneumococcal NA in this or a similar orientation (data not shown). Additionally, for the other three compounds active on viral NA (i.e., **1**, **7**, and **8**) we were unable to come up with a consistent and plausible binding mode. One reason could be the remarkable flexibility of NAs that include the regions of the binding site (Russell et al., 2006; Amaro et al., 2007, 2009; von Grafenstein et al., 2013). On the other hand, the inconsistent results suggest that this type of compound most likely binds in different orientations to the substrate binding site that are defined by ionic interactions with the active site arginines.

Enzyme kinetics experiments were conducted to investigate the binding mechanism of **1** (Table 2). Nonlinear regression and curve fitting modeled by SigmaPlot suggested a mixed-type inhibition for this compound on viral NA and all NanA constructs (Supplementary Figures 1, 2). Mixed-type inhibition indicates that the formation of the enzyme-substrate complex is inhibited as well as the turnover in case an enzyme-substrate complex is formed. Pre-incubation of viral NA with the inhibitor did not lead to lower *K*_i_ values, suggesting that **1** binds rapidly to the target. Thus, in contrast to approved NAIs (Barrett et al., 2011), **1** represents a fast-binding inhibitor. However, the fact that the intersection point of the different concentrations in the Lineweaver-Burk plots (Supplementary Figures 1, 2) is above the x-axis can be understood to mean that the affinity to the enzyme is higher than to the enzyme substrate complex. Thus, the results from the enzyme kinetics experiments are in agreement with the hypothesis that diazenylaryl sulfonic acids directly interact with the positively charged substrate binding site.

The importance of the ionic interaction is partially reflected in the structure-activity relationship of the compounds. The most active compound, **1**, also contains the highest number of sulfonic acid groups (i.e., four). Compound **5** differs from **4** by only one additional sulfonic acid group and is more active. However, also **8** differs from **7** by only one additional sulfonic acid group, and the activities of both compounds on viral and bacterial NA are comparable. Thus, the relationship between the number of charged groups (i.e., sulfonic acids) and activity on viral or pneumococcal NA is not consistent. Also, the presence of a sulfonic acid group is not sufficient for the biological activity of the investigated diazenylaryl sulfonic acids. For example, **9** contains three sulfonic acid groups and showed no or very low activity on any of the tested NAs.

Care must be taken during the quantitative interpretation of cell-based antiviral assays (Table 4). In general, IC_50_ values are expected to be higher for these more complex assays. Against expectation, compounds **4** and **7** showed an inhibition of the cytopathic effect (CPE) at concentrations that did not effectively inhibit neuraminidase activity. Similarly, **2** and **3** inhibited the CPE of two investigated influenza viruses with <15 μM concentration (Table 4), while NA activity was not inhibited at concentrations up to 100 μM or inhibited by 50% at higher concentrations, respectively (Table 1). Several azo compounds adhere to erythrocytes (as seen in the experimental controls of the HA assay; Supplementary Table 1), which can be disadvantageous for drug discovery. Taken together, these observations indicate that a further mechanism could be involved in mediating the anti-influenza activity of some diazenylaryl sulfonic acids.

The potent dual activity of **1** could be shown in NA inhibition assays as well as cellular experiments in the presence of virus and exogenous NanA (Figure 2), wherein the synergistic effect of the pneumococcal co-infection is simulated (McCullers and Bartmess, 2003). Given its good antiviral and antipneumococcal activity, its favorable cell compatibility, and the absence of hemagglutination, compound **1** could serve as an interesting starting point for the development of dual-acting NAIs. We recently identified such a dual inhibition mode for isoprenylated natural compounds including artocarpin, sanggenol A and congeners of sanggenol A (Walther et al., 2015; Grienke et al., 2016). Like diazenylaryl sulfonic acids, these natural compounds exert a stronger inhibitory effect on pneumococcal as compared to viral NA. However, some of the diazenylaryl sulfonic acids are stronger inhibitors of viral NA and viral replication than the natural compounds. In contrast, their antibacterial effect is weaker and bacteriostatic, whereas artocarpin, sanggenol A and its congeners act bactericidal and kill *S. pneumoniae*.

The strong effect of **1** on plaque formation (Figure 3) upon pretreatment of the virus is an indication that the activity of diazenylaryl sulfonic acids goes beyond that of the substrate-analogous inhibitors.

In summary, we characterized 17 diazenylaryl sulfonic acids regarding their anti-NA, antiviral and antibacterial activity, and cytotoxicity. Compound **1** unfolded as the most active compound, with *K*_i_ values <1.5 μM (FL assay) for both viral and pneumococcal NAs. It showed antiviral and antipneumococcal activity, and hence could serve as a starting point for the development of novel, dual-acting anti-infectives. All observations made with *in silico* and *in vitro* methods point toward diazenylaryl sulfonic acids binding to viral and pneumococcal NAs not in a purely competitive manner but in a mixed mode. A major component of activity can be ascribed to specific interaction with NAs, as evidenced by distinct bioactivity patterns observed for viral and pneumococcal NAs.

## Author contributions

JR, KL, MS, and JK conceived the work. SvG, CK, KL, and JK conducted the computational studies. SvG, JR, KL, MS, and JK selected the compounds for testing. AH, MR, EW, ZX, LS, and MS performed the antiviral tests, and UG, CM, and JR conducted the analytical and physicochemical studies. All authors contributed to the interpretation of the data and the writing of the manuscript. All authors have given approval to the final version of the paper.

## Funding

This work was funded by research grants P23051 and P24587 of the Austrian Science Fund (FWF), and research grant 2011FGR0137 of the Thuringian Ministry of Economy, Labor and Technology.

### Conflict of interest statement

The authors declare that the research was conducted in the absence of any commercial or financial relationships that could be construed as a potential conflict of interest.

## Footnotes

^1^*National Cancer Institute Developmental Therapeutics Program Diversity Set*. Available online at: https://dtp.cancer.gov/organization/dscb/obtaining/available_plates.htm (Accessed Oct 26, 2016).

^2^*National Cancer Institute Developmental Therapeutics Program database*. Available online at: http://dtp.nci.nih.,gov/docs/3d_database/Structural_information/structural_data.html (Accessed Aug 24, 2009).

## Abbreviations

BHI: brain heart infusion
CPE: cytopathic effect
DAD: diode array detector
DANA: 2,3-dehydro-2-deoxy-N-acetylneuraminic acid
DEMEM: Dulbecco's Modified Eagle Medium
DMSO: dimethyl sulfoxide
DTP: Developmental Therapeutics Program
EMEM: Eagle's minimum essential medium
ESI: electrospray ionization interface
FC: fold change
FL: fluorescence
HA: hemagglutination
HPLC: high performance liquid chromatography
IAV: influenza A virus
IPTG: isopropyl β-D-1-thiogalactopyranoside
LC: liquid chromatography
NA: neuraminidase
NanA: *S. pneumoniae* neuraminidase
NAI: neuraminidase inhibitor
NCI: National Cancer Institute
NIH: National Institute of Health
MD: molecular dynamics
MDCK: Madin-Darby canine kidney
MIC: minimal inhibitory concentration
MOI: multiplicity of infection
MS: mass spectrometry
MUNANA: 2′-(4-methylumbelliferyl)-α-D-N-acetylneuraminic acid sodium salt hydrate
OD: optical density
PAINS: pan assay interference compounds
PCR: polymerase chain reaction
PDB: Protein Data Bank
PFU: plaque-forming unit
RFU: relative fluorescence unit
rNanA: Recombinant *S. pneumoniae* neuraminidase
SD: standard deviation
TCID_50_: 50% tissue culture infective dose
TLC: thin-layer chromatography
TSB: tryptic soy broth
vis: visible
WT: wild type.
