# Supplementary material for: Discovery and Characterization of Diazenylaryl Sulfonic Acids as Inhibitors of Viral and Bacterial Neuraminidases
Source: Front Microbiol. 2017 Feb 15;8:205. doi: 10.3389/fmicb.2017.00205 (PMC5309245; doi:10.3389/fmicb.2017.00205)
Supplement: Supplementary file 1 [file DataSheet1.pdf]

Supplementary Material

Discovery and Characterization of Diazenylaryl Sulfonic Acids as Inhibitors of Viral and Bacterial Neuraminidases

Anja Hoffmann, Martina Richter, Susanne von Grafenstein, Elisabeth Walther, Zhongli Xu[1], Lilia Schumann, Ulrike Grienke, Christina E. Mair, Christian Kramer, Judith M. Rollinger, Klaus R. Liedl, Michaela Schmidtke, Johannes Kirchmair[*]

*** Correspondence:** Johannes Kirchmair: kirchmair@zbh.uni-hamburg.de

1 Supplementary Figures and Tables

1.1 Supplementary Figures

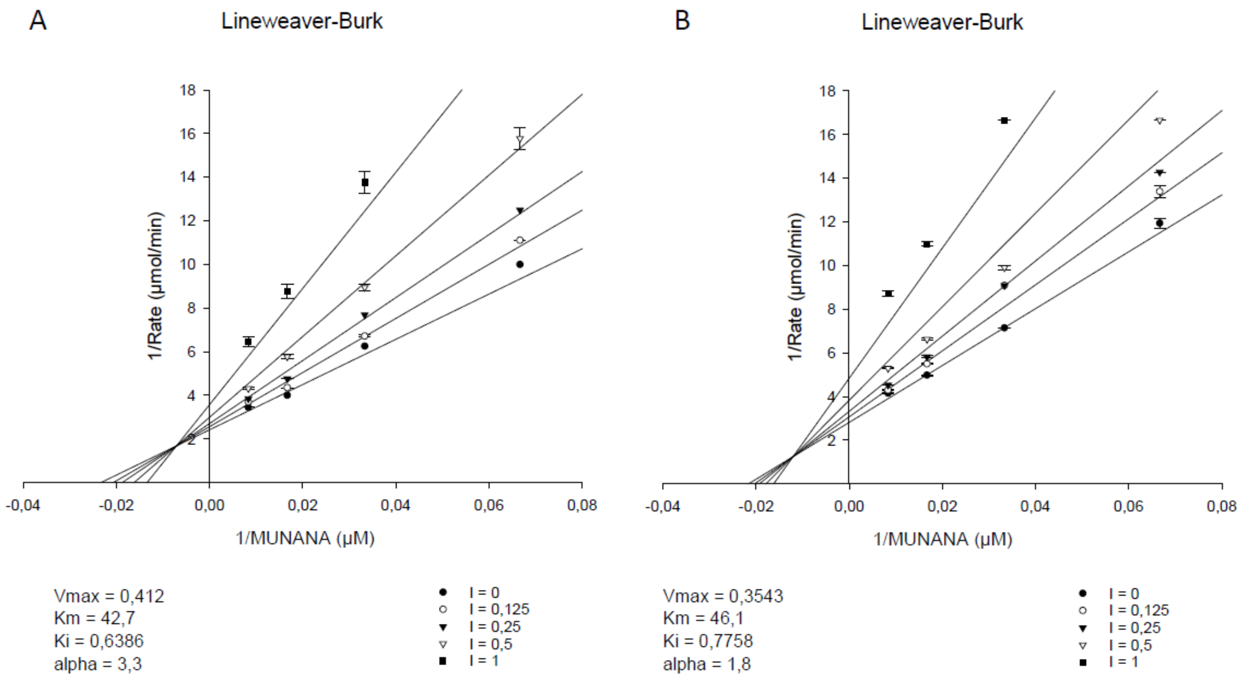

Supplementary Figure 1. Lineweaver-Burk plots of the enzyme kinetics influenza virus A/WSN/33 with **1**. (A) Virus was preincubated for 20 min with **1**. (B) Virus, **1**, and MUNANA were co-incubated without a pre-incubation step. The tested concentrations are shown in the legend box of each graph.

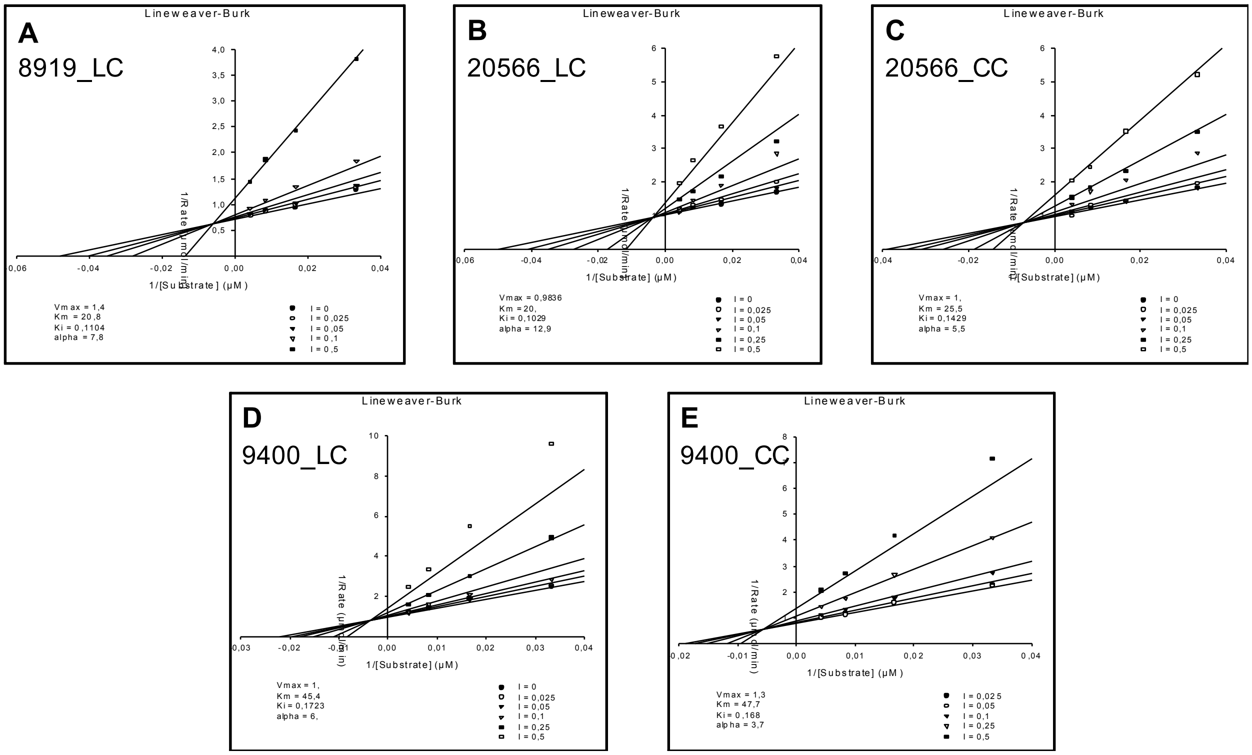

Supplementary Figure 2. Lineweaver-Burk plots of the enzyme kinetics of NanA with **1**. Each NanA construct was tested in three independent assays at concentrations of **1** shown in the legend box of each graph.

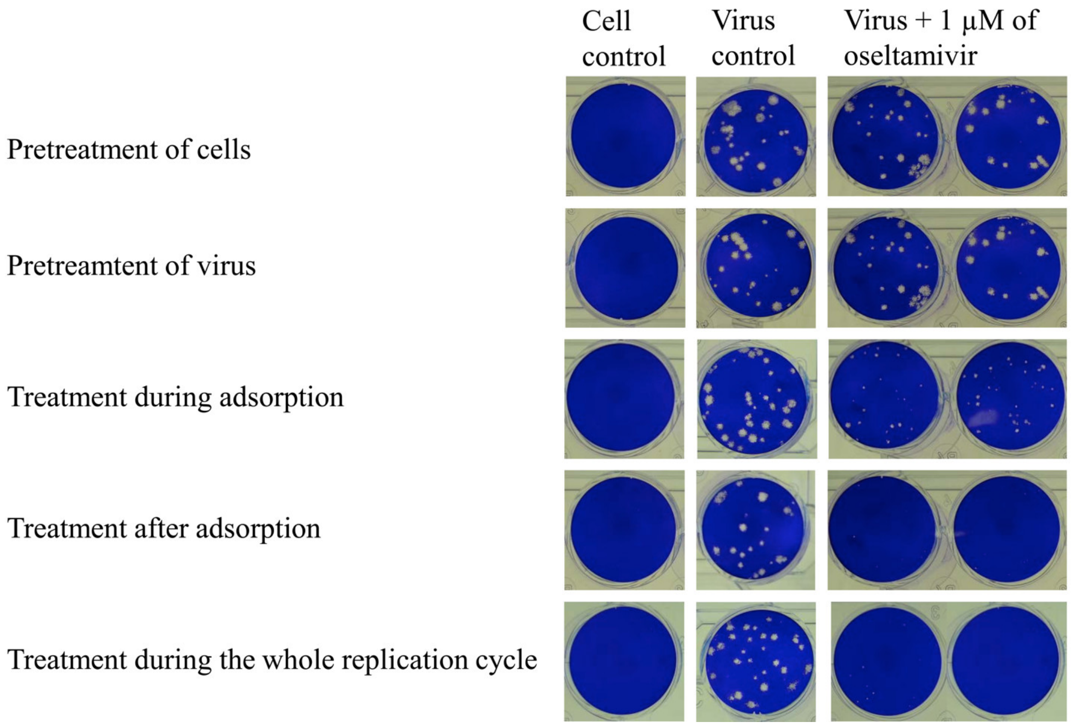

Supplementary Figure 3. Plaque size reduction observed after treatment with 1 µM of oseltamivir.

1.2 Supplementary Tables

Supplementary Table 1. Assay interference phenomena of test compounds in the HA-based assay.

Cpd.	sample concentrations [μM] in viral HA-assay provoking hem-agglutination[a]	prevention of virus-induced hemagglutination[b]	erythrocyte lysis[c]	bacterial HA-assay provoking hem-agglutination[d]	erythrocyte lysis[e]
**1**	no	no	no	no	no
**2**	no	3.16-100	no	no	no
**3**	3.16-100	no	no	31.6-100	no
**4**	1-100	no	no	no	100
**5**	no	31.6-100	no	no	no
**6**	100 or no	no	no	no	no
**7**	1-100	no	no	no	no
**8**	no	no	no	no	no
**9**	no	no	no	no	no
**10**	100 or no	no	no	no	no
**11**	no	no	no	no	no
**12**	no	no	no	no	no
**13**	no	no	no	no	no
**14**	31.6-100	no	no	no	no
**15**	100 or no	no	no	no	no
**16**	no	no	no	no	no
**17**	no	no	no	no	no

[a]Test compound induced a hemagglutination of human erythrocytes (2 h at 4°C).
[b]Test compound prevented the virus-induced hemagglutination of human erythrocytes (2 h at 4°C).
[c]Test compounds lysed the human erythrocytes (2 h at 4°C).
[d]Test compound induced a hemagglutination of human erythrocytes in the presence of lectin (24 h, 4°C).
[e]Test compounds lysed the human erythrocytes (4 h at 37°C and 12 h at 4°C).

Supplementary Table 2. Virus titers (plaque-forming units, PFU) and reduction in plaque numbers by treatment with **1**.

Experiment	Untreated virus control titer (PFU/ mL)	rNanA *S. pneumoniae* DSM20566	Virus titer after treatment with 10 µM of **1** (PFU/ mL)	Reduction in plaque numbers (%)	Mean reduction in plaque numbers ± SD (%)
I	14 750	-	900	93.90	94.12 ± 3.06
			1 300	91.19	
			400	97.29	
	20 500	+	1 300	93.66	96.10 ± 3.45
			300	98.54	
II	11 167	-	480	95.70	96.46 ± 1.08
			310	97.22	
	20 750	+	560	97.30	97.76 ± 0.65
			370	98.76	
**Mean ± SD of both experiments**		**In the absence of pneumococcal NA**			**95.29 ± 1.65**
		**In the presence of pneumococcal NA**			**96.93 ± 1.17**

Supplementary Table 3. Effect of **1** and **2** on *S. pneumoniae* in comparison to the control antibiotic imipenem.

Compound	Tested compound concentration (µM)	cfu per agar plate	Number of replicates	Activity
Imipenem	0.1	< 150	3	bactericidal
**1**	50	> 150[a]	2	bacteriostatic
**2**	50	> 150[a]	2	bacteriostatic
	25	> 150[a]	2	bacteriostatic

[a]Abundant bacterial growth. The exact number of colony forming units was not determined.
